# Eligibility for Lung Cancer Screening Among Women Receiving Screening for Breast Cancer

**DOI:** 10.1001/jamanetworkopen.2022.33840

**Published:** 2022-09-30

**Authors:** Ashley L. Titan, Ioana Baiu, Doug Liou, Natalie S. Lui, Mark Berry, Joseph Shrager, Leah Backhus

**Affiliations:** 1 Department of Surgery, Stanford University Hospital, Stanford, California; 2 Department of Cardiothoracic Surgery, Stanford University Hospital, Stanford, California; 3 Stanford School of Medicine, Stanford, California

## Abstract

Blurb: This cohort study assesses the number of women who were eligible for and underwent lung cancer screening among those who received mammograms at a single academic medical center.

## Introduction

The US Preventive Services Task Force (USPSTF) recommends mammography for breast cancer screening (BCS) annually for women older than 40 years with a family history and biennially for women aged 50 to 74 years.^1^ More than 70% of eligible women undergo BCS.^2^ Meanwhile, the screening rate for lung cancer, the leading cause of cancer-related death, is comparatively low among eligible women.^3^ Annual lung cancer screening (LCS) recommendations were updated in 2021 to include high-risk individuals aged 50 to 80 years with a smoking history of at least 20 pack-years, those who currently smoke, and those who have quit within the past 15 years.^3^ These criteria were largely based on the National Lung Screening Trial results, which demonstrated a 20% reduction in mortality from lung cancer (LC) among high-risk women who underwent low-dose computed tomography compared to chest radiography.^4^ In a recent survey, one-half of primary care physicians (PCPs) were not familiar with USPSTF recommendations.^5^ Apart from the lack of education and awareness regarding LCS, screening barriers, such as nihilism among clinicians and anxiety over a potential diagnosis among women remain pervasive.^6^ Screening for LC remains underused in women. The aim of this study was to assess the number of with a smoking history who underwent BCS women at a single academic medical center and were also eligible for LCS.

## Methods

This retrospective, single-center cohort study included women aged 55-74 years who underwent BCS at Stanford University Hospital between January 2019 and June 2020 and had a smoking history. This study followed the STROBE reporting guideline and was approved by the Stanford University Institutional Review Board, which waived the requirement for informed consent because only deidentified data were used.

We hypothesized that most eligible women do not undergo LCS, and PCPs would preferentially order BCS more than LCS. The primary outcome was the number of women who received mammograms for BCS who were also eligible for LCS using low-dose computed tomography. Secondary outcomes were the number of women diagnosed with LC and the clinician ordering LCS. The 2013 USPSTF criteria were used to determine eligibility for LCS. Statistical analysis was performed using χ^2^ test and a multivariable cox proportional hazards model adjusted for confounders (age, race, employment status, insurance status, personal history of pulmonary conditions, family history of LC, pack years, and years since quitting smoking). Significance was set at *P* < .05 and analysis was performed using Stata 15 (Stata Corp, LLC).

## Results

Of 874 women (mean [SD] age, 66.8 [5.4] years) who underwent BCS, 99 (11.3%) were eligible for LCS, but only 35 (35.5%) were screened. The proportion of eligible women who were diagnosed with LC using the 2013 USPSTF criteria was significantly greater than in ineligible women (6.0% vs 2.5%, *P* = .02, Table 1). Among those eligible based on the updated guidelines, the difference was even greater (8.0% vs 1.8%, *P* = .007). Cancer screening was ordered by PCPs in 82.6% of women eligible for BCS and 60.0% of those eligible for LCS (*P* < .001, Table 2). Eligibility status for LCS was not associated with increased likelihood of undergoing LCS.

**Table 1.** Participant Demographic Characteristics

Characteristic	Participants, No. (%)			*P* value
	Total	Eligible	Ineligible	
Participants	874 (100)	99 (11.3)	775 (88.7)	
Age, mean (SD)	66.8 (5.4)	67.4 (5.1)	66.7 (5.4)	
Race^a^				
Asian	70 (8.0)	3 (3.0)	67 (8.6)	.04
Black	53 (6.1)	11 (11.1)	42 (5.4)	
Native American	9 (1.0)	5 (5.1)	4 (0.5)	
Pacific Islander	6 (0.7)	0	6 (0.8)	
White	633 (72.4)	71 (71.7)	562 (72.5)	
Other^b^	96 (11.0)	8 (8.1)	87 (11.2)	
Unknown	8 (0.9)	1 (1.0)	7 (0.9)	
Family history				
Cancer	654 (74.8)	65 (65.7)	589 (76.0)	.08
Breast, ovarian, or uterine cancer	389 (44.5)	39 (39.4)	350 (45.2)	.47
Lung cancer	110 (12.6)	12 (12.1)	98 (12.6)	.85
Insurance status				
Insured	766 (87.6)	88 (88.9)	684 (88.3)	.69
Unknown insurance status	108 (12.4)	11 (11.1)	91 (11.7)	
Screening				
LCS	73 (8.4)	35 (35.4)	0	.01
No LCS	502 (57.4)	36 (36.4)	466 (60.1)	
Chest CT for another reason	299 (34.2)	28 (28.3)	309 (39.1)	
Lung cancer diagnosis	25 (100)	6 (6.1)	19 (2.5)	
Stage I disease	19 (76.0)	4 (66.7)	14 (73.7)	.02

Abbreviations: CT, computed tomography; LCS, lung cancer screening.

^a^ Data on race were self-reported by the participants.

^b^ Other race was undefined.

**Table 2.** Clinicians Ordering Lung Cancer Screening for Eligible Participants

Specialty	Clinicians, No. (%)		*P* value
	Ordered mammogram	Ordered LCS	
Primary care	30 (85.7)	21 (60.0)	<.001
Pulmonology	0	10 (28.6)	
Obstetrics and gynecology	3 (8.6)	0	
Other^a^	2 (5.7)	4 (11.4)	

^a^ Other includes the following specialties: oncology, surgical oncology, and thoracic surgery.

## Discussion

Despite being proven to decrease mortality, LCS is underused among eligible women, in contrast to breast and colorectal cancer screening.^1^ Identifying women eligible for LCS during their BCS could increase detection rates and potentially at an earlier stage. This finding is consistent with previous literature and highlights the potential benefit of pairing BCS with effective strategies for LCS.^3^

The data suggest the same clinicians who follow BCS guidelines fail to follow LCS guidelines, possibly leading to decreased screening rates in eligible women. There is an opportunity for increased education among PCPs and patients to help with the shared decision-making about LCS. Limitations of this study include its retrospective nature, small cohort from a single institution, and inability to identify why eligible patients did not undergo LCS. Education regarding indications and importance of LCS is paramount to increasing its use.
